# Improved accuracy of etiological diagnosis of spinal infection by metagenomic next-generation sequencing

**DOI:** 10.3389/fcimb.2022.929701

**Published:** 2022-10-07

**Authors:** Liang Xu, Zheng Zhou, Yao Wang, Chao Song, Hongdong Tan

**Affiliations:** ^1^ Department of Spinal Infection, Shandong Public Health Clinical Center, Jinan, China; ^2^ Katharine Hsu International Research Institute of Infectious Disease, Shandong Public Health Clinical Center, Jinan, China; ^3^ State Key Laboratory of Translational Medicine and Innovative Drug Development, Jiangsu Simcere Diagnostics Co., Ltd., Nanjing, China; ^4^ Department of Medicine, Nanjing Simcere Medical Laboratory Science Co., Ltd., Nanjing, China

**Keywords:** metagenomic next-generation sequencing, microbiological test, spinal infection, diagnostic accuracy, antimicrobial therapy

## Abstract

Currently, the use of metagenomic next-generation sequencing (mNGS), a new approach to identify organisms in infectious diseases, is rarely reported in the diagnosis of spinal infection. This study aimed to evaluate the potential value of mNGS in etiological diagnosis of spinal infection. In this retrospective study, the clinical data of patients with suspected spinal infection were collected by electronic medical records. Specimens obtained from each patient were tested *via* mNGS assay and other conventional microbiological tests (CMTs). The sensitivity and specificity of mNGS and CMTs were calculated using the final clinical diagnosis as the golden standard. In total, 108 patients were eligible for the study, with the mean length of stay of 42.8 days. Regarding the overall identification of pathogens, mNGS exhibited a better performance than CMTs, and several nontuberculous mycobacteria, fungi, and bacteria were newly discovered. In the diagnosis of spinal infection, the sensitivity, specificity, and area under the curve of mNGS were 90.72%, 81.82%, and 0.89, respectively, which were all higher than 52.17%, 56.25%, and 0.72 of the CMTs. At hospital discharge, the C-reactive protein, erythrocyte sedimentation rate, and white blood cell count of patients significantly decreased compared with hospitalization (all *p* < 0.05), and 88.89% showed good outcomes. These findings may suggest that mNGS has a better diagnostic accuracy in pathogenic identification of patients with suspected spinal infection, and patients treated with NGS-guided antimicrobial therapy mostly seem to have good outcomes.

## Introduction

Spinal infections, which commonly occur as vertebral osteomyelitis, discitis, and epidural abscesses, have the potential to cause significantly serious consequences even death if not treated promptly and precisely at early stage, although the overall incidence is not very high (approximately 2.2/100,000 per year) (Beronius et al., 2001; Grammatico et al., 2008; Duarte and Vaccaro, 2013). Because of aging population and the increased number of immunocompromised people in China, the incidence of spinal infections is increasing year by year. However, the onset of this disease is insidious, and the performance is not typical, causing a great trouble to the diagnosis (Zhang et al., 2019). A significant delay of approximately 2–6 months usually occurs until establishment of a final diagnosis and treatment because of the low specificity of signs and symptoms at clinical presentation (Lener et al., 2018).

As an innovative etiological diagnostic tool, metagenomic next-generation sequencing (mNGS) technology has been widely used in the diagnosis of various infectious diseases with advantages of rapidly and unbiasedly capturing pathogens as well as species identification in a single clinical sample (Simner et al., 2018). Notably, this non-targeted identification strategy is revolutionizing the field of microbial diagnostics away from conventional diagnostic strategies based on prior assumptions regarding some certain pathogens (Besser et al., 2018). Another benefit of mNGS is the shortened turnaround time of about 30 h, which overcomes the difficulty of long cultivation period for some microorganisms, such as mycobacteria, and provides more possibilities for early and effective treatment (Li et al., 2021). Consequently, clinicians of infectious department have used mNGS frequently for detection of pathogens in various clinical specimens, including blood, cerebrospinal fluid, and bronchoalveolar lavage fluid (Wilson et al., 2014; Parize et al., 2017; Wilson et al., 2018; Miller et al., 2019; Qian et al., 2021). Indeed, mNGS provides an alternative to diagnose patients with suspected infections for which the pathogenic cause is unknown despite prior comprehensive microbiological investigations.

Currently, there are few studies on the use of mNGS in the identification of pathogens within spinal infection-associated tissue specimens. Therefore, this study was performed to evaluate the potential value of mNGS in the etiological diagnosis of spinal infection.

## Materials and methods

### Patients

In this study, the patients with suspected spinal infection from Shandong Chest Hospital between March 2020 and August 2021 were retrospectively analyzed. Inclusion criteria included are as follows: (1) fever; (2) presence of chest pain not relieved by rest or analgesics; (3) abnormal magnetic resonance imaging; and (4) previous and current episodes of tuberculosis (TB). Patients with incomplete clinical information were excluded. Written consent forms were obtained from all the patients. This study was approved by the Institutional Review Board of Shandong Chest Hospital (approval number: 2021XKYYEC-44), a part of Shandong Public Health Clinical Center.

### Clinical data collection

The clinical data of patients were collected by directly reviewing electronic medical records in hospital system, including gender, age, infectious sites, comorbidities, clinical symptoms, laboratory results, radiological features, and treatment regimens.

### Specimen collection and conventional microbiological tests

**Figure 1:**
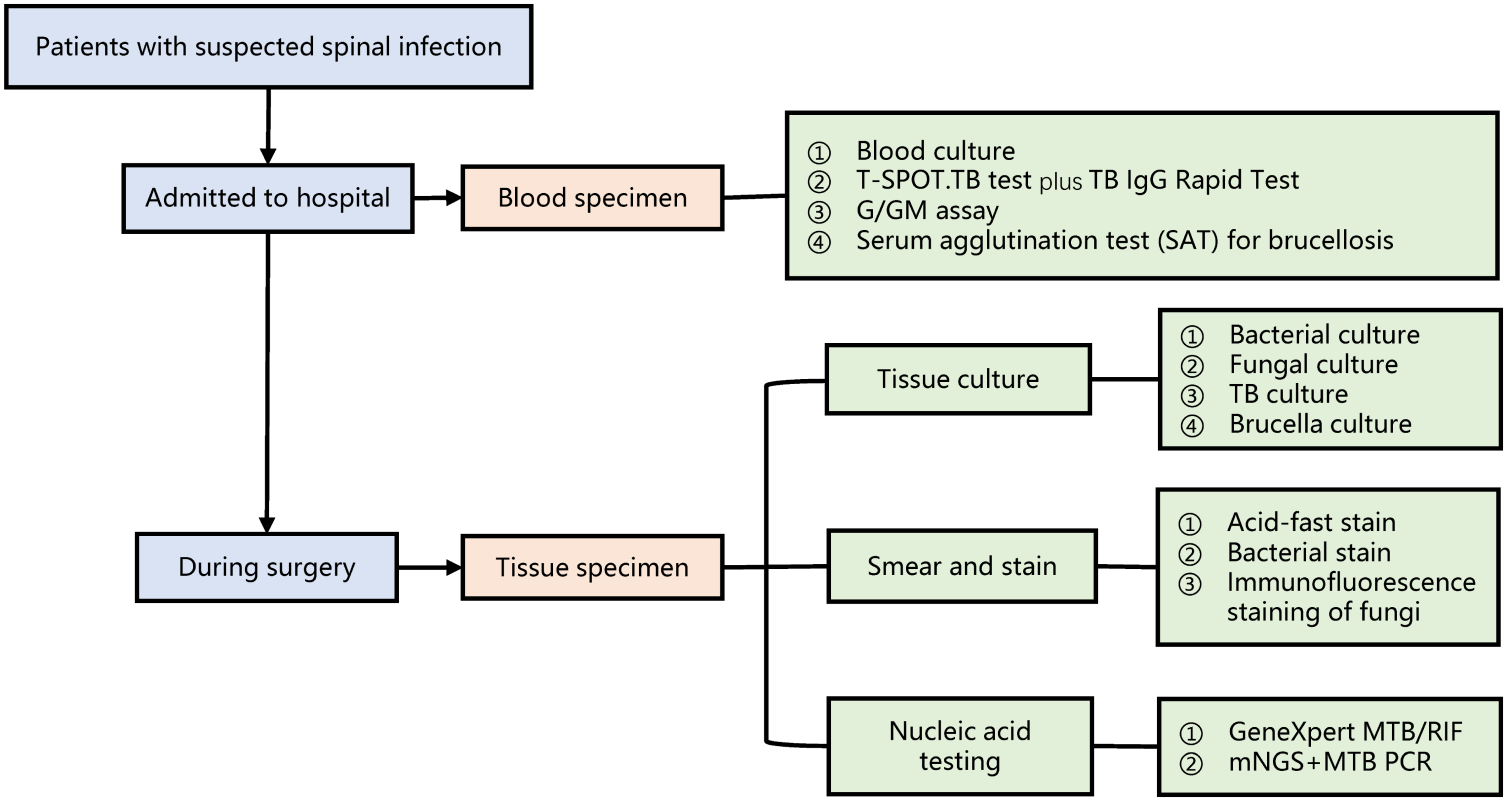
The workflow of specimen collection and test methods possibly involved in the patients.

When suspected spinal infection was diagnosed, blood specimens were obtained immediately and tested by four conventional methods, including blood culture, T-SPOT.TB test plus TB immunoglubin G (IgG) rapid test, G/GM assay, and serum agglutination test for brucellosis. Tissue specimens were achieved through surgical biopsy and submitted to clinical laboratories for pathogenic culture, smear and staining, and nucleic acid testing (X-pert and mNGS). The workflow of specimen collection and test methods possibly involved in the patients was presented in **Figure 1**.

### Metagenomic next-generation sequencing

Tissue samples were processed for wall breaking through an in-house developed method. The tissue was first transferred into a lysis tube (MP Lysing Matri A), 400 μl of phosphate-buffered saline was added, and then the MP FastPrep-24™ 5G instrument was used for shaking at 6 m/s for 120 s. After wall breaking, nucleic acid extraction was conducted using a micro-sample genomic DNA extraction kit (DP316, Tiangen), and the nucleic acid extracted was subjected to nucleic acid quantitative quality inspection using Qubit 4.0. The nucleic acid that passed the quality inspection participated in the process of library construction as follows: the DNA was first cut into about 200 bp by enzyme digestion; then, end-repair, a-tail adjunction, adapter ligation, and labeling were performed; finally, polymerase chain reaction (PCR) was applied for enrichment, and Agilent 4200 was used to detect the fragments of the library. Subsequently, the library concentration quality was checked using Qubit 4.0. The library with qualified fragments and concentration was sequenced on an Illumina NextSeq 550 DX platform for single-end 75-bp sequencing.

Fastp was used for FASTQ file quality control. High-quality sequencing data were generated by removing low-quality reads, adapter contamination, short reads (50 bp), and duplicated reads. Human sequence data were excluded and mapped to a human reference (GRCh38) using a powerful alignment tool called Burrows–Wheeler Alignment (Li and Durbin, 2009). After removing human sequences, the remaining sequencing data were aligned to National Center for Biotechnology Information nucleotide (NCBI nt) database by Scalable Nucleotide Alignment Program (Zaharia et al., 2011). As described in a previous study (Chen et al., 2020), the mapped data were processed with in-house scripts, including taxonomy annotation, genome coverage/depth calculation, and abundance calculation. Kraken2 was used to perform microbial classification. Abundance of species within a sample was estimated with Braken. The results were further verified by BLAST.

### Clinical golden standards and outcome assessment

In this study, the clinical golden standard was described as the final clinical diagnosis, which was decided by physicians based on the patient’s clinical records, microbiological tests, imaging and histopathological analysis, past medication information, etc. The sensitivity and specificity were calculated on the basis of this.

Physicians adjusted the treatment strategy according to mNGS results. To evaluate the effect of the mNGS-guided treatment, three representative inflammatory indicators—white blood cell count (WBC), erythrocyte sedimentation rate (ESR), and C-reactive protein (CRP)—were recorded and compared at the time of hospitalization and hospital discharge. The clinical outcomes were described as complete recovery, improved symptoms, voluntary hospital discharge, and death.

### Statistical analysis

All statistical analyses were performed using R package (version 3.6.3). The dichotomous variables were counted to evaluate the sensitivity and specificity of different detection methods, and the results were presented with 95% confidence intervals (CIs). R package “pROC” (version 1.18) was employed to draw receiver operator characteristic (ROC) curves. The Wilcoxon rank sum test was used to compare the differences of three inflammatory indicators before and after treatment under the mNGS-guided therapy. The value of *p* < 0.05 was considered statistically significant.

## Results

### Patient characteristics

Between March 2020 and August 2021, 108 patients were eligible for the study, among whom 55 were male patients. Their mean age was 57.8 years, and the mean length of stay was 42.8 days. The infectious sites included lumbar spine (75.9%), thoracic spine (18.5%), lumbar and thoracic spine (4.6%), and cervical spine (0.9%). Among 108 cases, 103 (95.4%) experienced the pain at the infected sites, and 47 (43.5%) reported limb pain and numbness prior to the hospital admission. Fever or recurrent fever occurred in 34.3% of patients before or during hospitalization. In addition, 5.6% of patients experienced weight loss and another 3.7% had night sweats. The most frequent comorbidities were diabetes (20.4%) and hypoalbuminemia (20.4%). **Table 1** describes the patient characteristics in detail.

**Table 1:** Characteristics of 108 patients enrolled in this study.

Characteristics	N (%)
Age, years, median (range)	57.8 (14.0–82.0)
Gender (male)	55 (58.5)
Length of stay, day, median (range)	42.8 (6.0–120.0)
Infectious sites	
Lumbar spine	82 (75.9)
Thoracic spine	20 (18.5)
Thoracic spine and lumbar spine	5 (4.6)
Cervical spine	1 (0.9)
Symptoms	
Pain	103 (95.4)
Limb pain and numbness	47 (43.5)
Fever	37 (34.3)
Weight loss	6 (5.6)
Night sweat	4 (3.7)
Laboratory parameters, median (range)	
WBC count, 10 × 9 cells/L	6.6 (2.7–12.5)
ESR, mm/H	51.7 (3.0–124.0)
CRP, mg/L	39.2 (0.1–165.5)
Comorbidities	
Diabetes	22 (20.4)
Hypertension	22 (20.4)
Liver and renal dysfunction	8 (7.4)
Immunosuppression	1 (0.9)
Others	36 (33.3)

WBC, white blood cell count; ESR, erythrocyte sedimentation rate; CRP, C-reactive protein.

### Isolates of pathogens identified by the clinical golden standard

**Figure 2:**
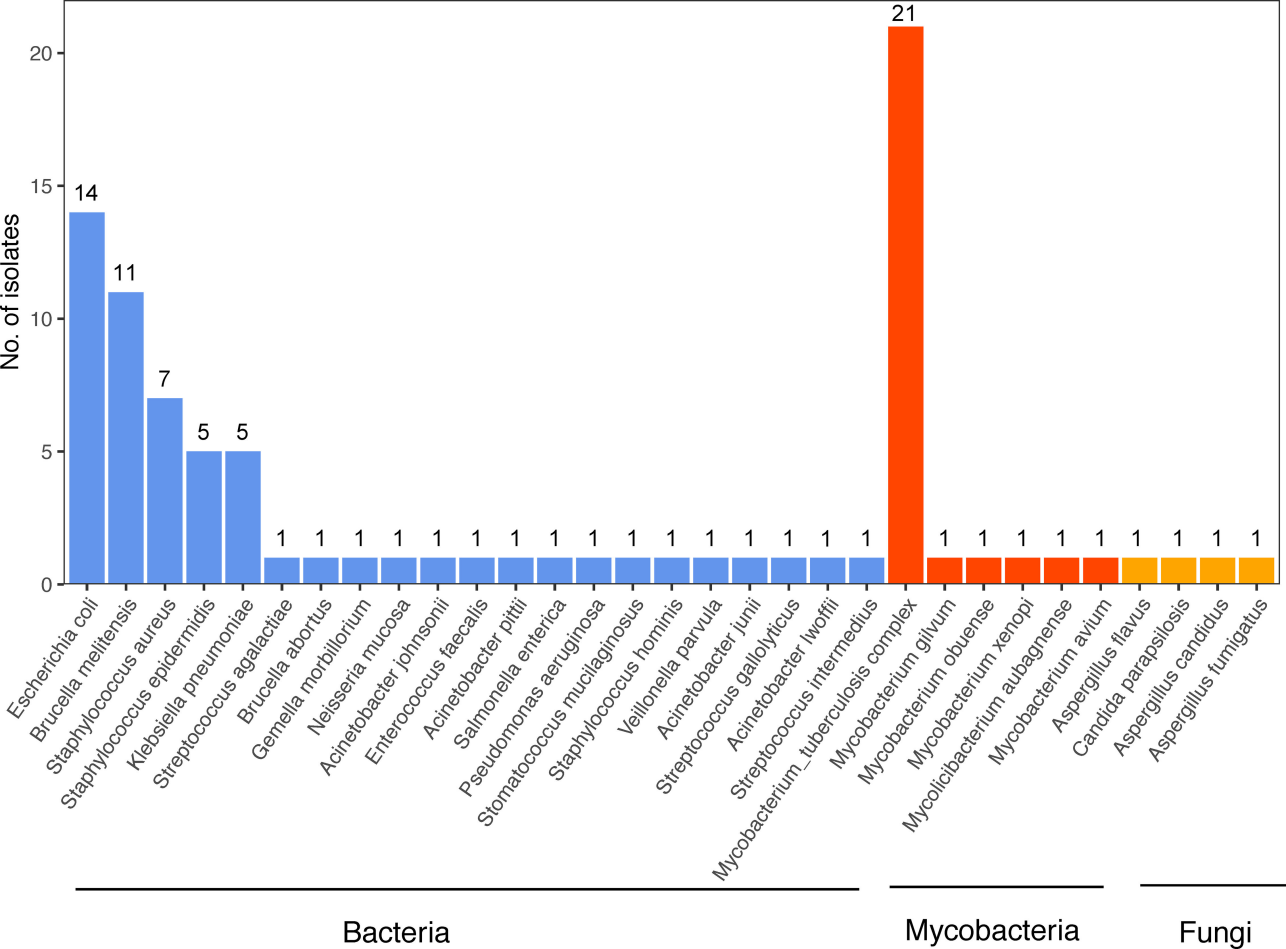
Isolates of pathogens detected by the clinical golden standard.

As shown in **Figure 2**, a total of 27 types of bacteria (including six types of mycobacteria) and four types of fungi were detected through the clinical golden standard. Among mycobacteria, *Mycobacterium tuberculosis* (MTB) was detected with the highest number of isolates (n = 21), and five non-tuberculous mycobacteria (NTM) were detected once each. Except for mycobacteria, the top five bacterial isolates were *Escherichia coli*, *Brucella melitensis*, *Staphylococcus aureus*, *Staphylococcus epidermidis*, and *Klebsiella pneumoniae*. The four fungi with one isolate included *Aspergillus flavus*, *Aspergillus candidus*, *Aspergillus fumigatus*, and *Candida parapsilosis*.

### Concordance between CMTs and mNGS

**Figure 3:**
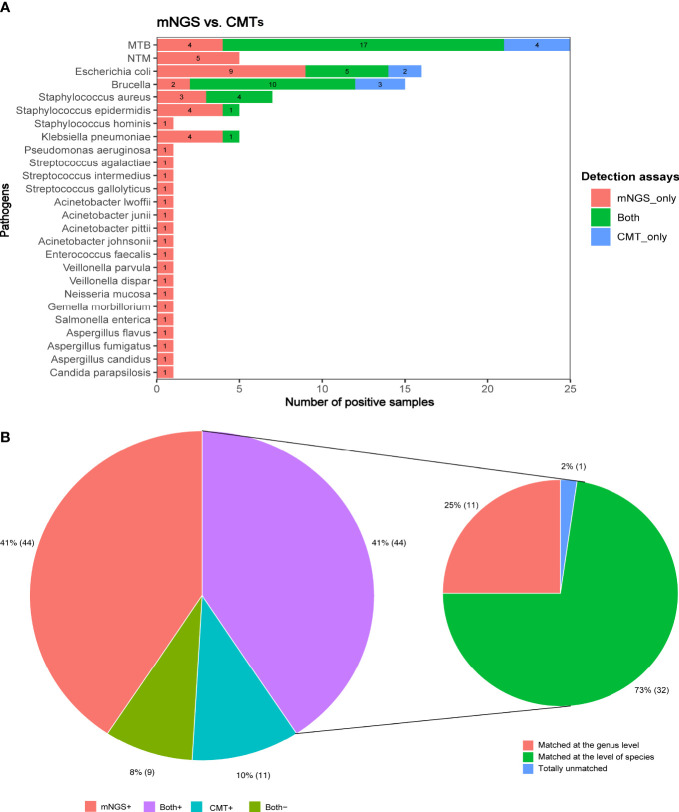
**(A)** The number of positive samples detected by CMTs and mNGS for each pathogen identified by the clinical golden standard. **(B)** The concordance between mNGS and CMTs in the detection of pathogens.

For the identification of MTB, mNGS and conventional microbiological tests (CMTs) showed the same detection ability. The MTB was identified in 17 cases by both mNGS and CMTs, in four cases only by mNGS, and four cases only by CMTs. Brucella was detected in two cases using mNGS alone and three cases using CMTs alone. Except for the pathogens mentioned above, mNGS exhibited a better detection performance than CMTs in all other pathogens. *Escherichia coli* was detected in nine cases by mNGS but only in two cases by CMTs. Moreover, *Staphylococcus aureus* (n = 3), *Staphylococcus epidermidis* (n = 4), and *Klebsiella pneumoniae* (n = 4) were missed by CMTs but detected by mNGS. Notably, novel pathogens were discovered by mNGS, including NTM (n = 5), fungi (n = 4), and bacteria (n = 15) (**Figure 3A**).

Of all patients, the pathogens were identified in 44 cases using both CMTs and mNGS assays, in 44 cases using mNGS alone, and in 11 cases using CMTs alone; no pathogens were detected in nine cases by either CMTs or mNGS assays (**Figure 3B**). Analysis of the pathogens detected in 44 patients using both CMTs and mNGS assays showed that the pathogens were completely matched in 32 cases at the level of species and only matched in 11 cases at the genus level but totally unmatched in one case.

### Diagnostic performance of mNGS

**Figure 4:**
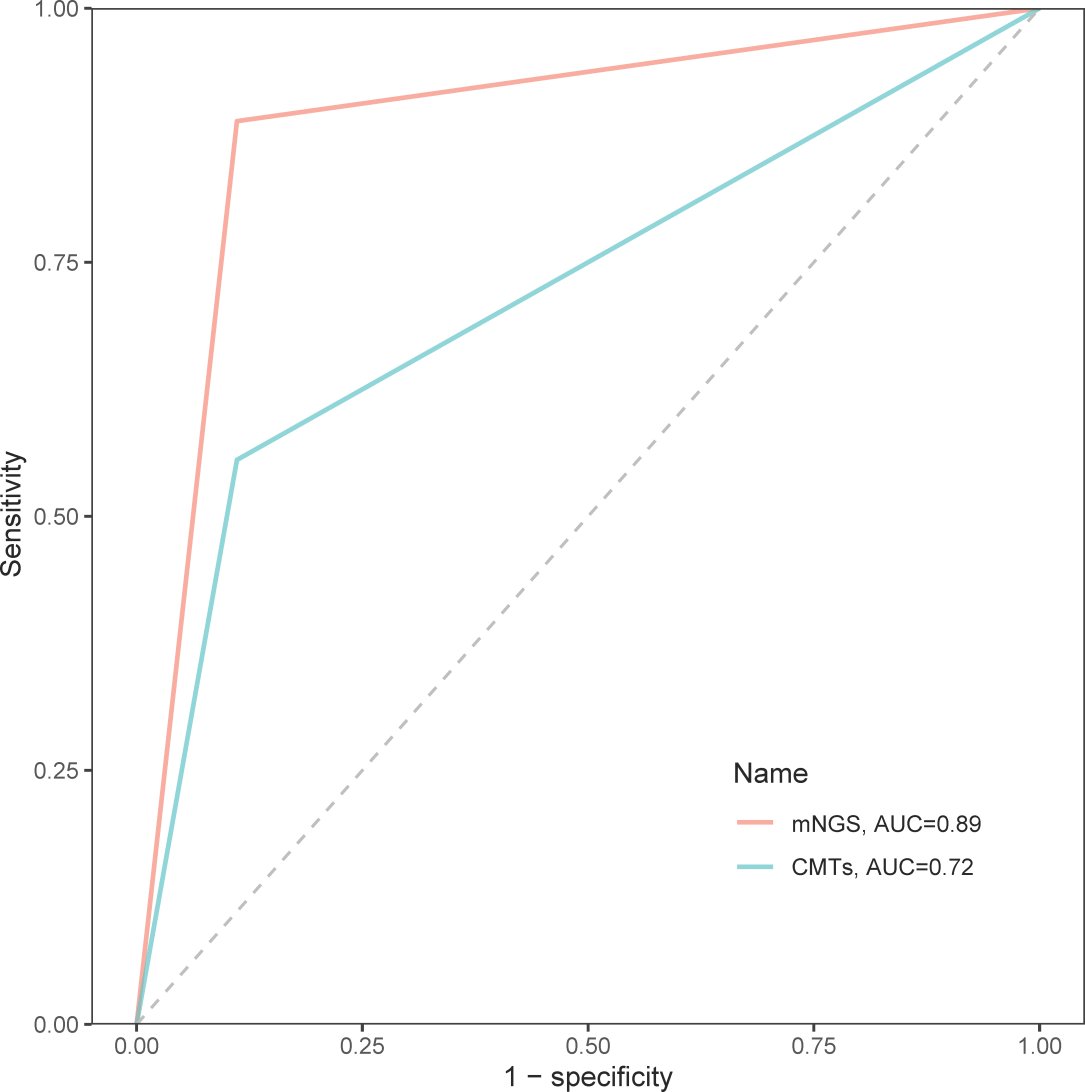
The receiver operator characteristic curves of mNGS and CMTs in the diagnosis of spinal infection.

In the diagnosis of spinal infection, the overall sensitivity of mNGS came up to 90.72%, significantly higher than 52.17% of the CMTs (*p* < 0.001), and its overall specificity was also slightly higher than that of CMTs although no statistical significance (81.82% vs. 56.25%, *p* = 0.231). Regarding the identification of bacteria, mycobacteria, and fungi, the sensitivity of mNGS was all superior to that of CMTs (bacteria: 90.30% vs. 53.40%; mycobacteria: 83.90% vs. 67.74%; fungi: 100.00% vs. 25.00%; **Table 2**). In addition, the area under the curve (AUC) of mNGS was 0.89, significantly larger than 0.72 of CMTs, which further confirmed a better diagnostic performance of mNGS than CMTs in spinal infection (**Figure 4**).

**Table 2:** Diagnostic performance of mNGS vs. CMTs in spinal infection.

Pathogens	Testing	Sensitivity (95% CI; n/N)	Specificity (95% CI; n/N)
All	mNGS	90.72% (0.827–0.954; 88/97)	81.82% (0.478–0.968; 9/11)
	CMTs	52.17% (0.416–0.626; 48/92)	56.25% (0.306–0.792; 9/16)
Bacteria	mNGS	90.30% (0.82–0.952; 84/93)	81.82% (0.478–0.968; 9/11)
	CMTs	53.40% (0.425–0.641; 47/88)	56.25% (0.306–0.792; 9/16)
Mycobacteria	mNGS	83.90% (0.655–0.939; 26/31)	90.00% (0.541–0.995; 9/10)
	CMTs	67.74% (0.485–0.826; 21/31)	90.00% (0.541–0.995; 9/10)
Fungi	mNGS	100.00% (0.396–1; 4/4)	100.00% (0.629–1; 9/9)
	CMTs	25.00% (0.013–0.78; 1/4)	100.00% (0.628–0.996; 9/9)

mNGS, metagenomic next-generation sequencing; CMTs, conventional microbiological tests.

### Inconsistent analysis between mNGS and CMTs

Mycobacteria in nine patients were detected by mNGS but missed by CMTs. Meanwhile, mycobacteria in four patients were identified by CMTs but missed by mNGS (**Figure 3A**). Some of these negative mNGS results may be due to the low concentration of nucleic acid extraction caused by the difficulty in the cell wall breaking of mycobacteria. There were also some cases confirmed by mNGS but false positive by CMTs because of contamination (**Table 3**). The fungi in four patients were identified by mNGS at the level of species but missed by CMTs, among which higher G/GM test scores related to possible fungi infection were suggested in three cases (**Table 4** **)**.

**Table 3:** Inconsistent analysis between mNGS and CMTs in the bacterial subgroup.

Patient no.	mNGS	PCR for MTB	T-spot	Culture	X-pert	Clinical golden standard	Comments
4	−	NaN	−	−	+	MTB	MTB missed by mNGS
6	−	+	+	MTB	+	MTB	
11	*M. Aubagnense*	−	+	−	−	M. Aubagnense	T-spot unable to distinguish MTB and NTM
12	*M. luteum*	−	+	−	−	*M. luteum*	
34	*E. coli*	−	−	*E. faecalis*	−	*E. coli*	Culture contaminated
60	*E. coli*	−	−	*S. epidermidis*	−	*E. coli*	

mNGS, metagenomic next-generation sequencing; CMTs, conventional microbiological tests; PCR, polymerase chain reaction; MTB, mycobacterium tuberculosis; NTM, non-tuberculous mycobacteria.

**Table 4:** Analysis of inconsistent results between mNGS and CMTs in fungi subgroup.

Patient no.	mNGS	G/GM	Culture	Clinical golden standard	Comments
4	*Aspergillus flavus*	+	Aspergillus	*Aspergillus flavus*	CMTs and mNGS results matched at the genus level.
6	*Aspergillus fumigatus*	+		*Aspergillus fumigatus*	Possible fungal infection suggested by CMTs was verified by mNGS.
78	*Aspergillus luteus*	+		*Aspergillus luteus*	
51	*Candida parapsilosis*			*Candida parapsilosis*	Pathogens missed by CMTs were detected by mNGS.

mNGS, metagenomic next-generation sequencing; CMTs, conventional microbiological tests.

### Outcomes under the mNGS-guided antimicrobial therapy

**Figure 5:**
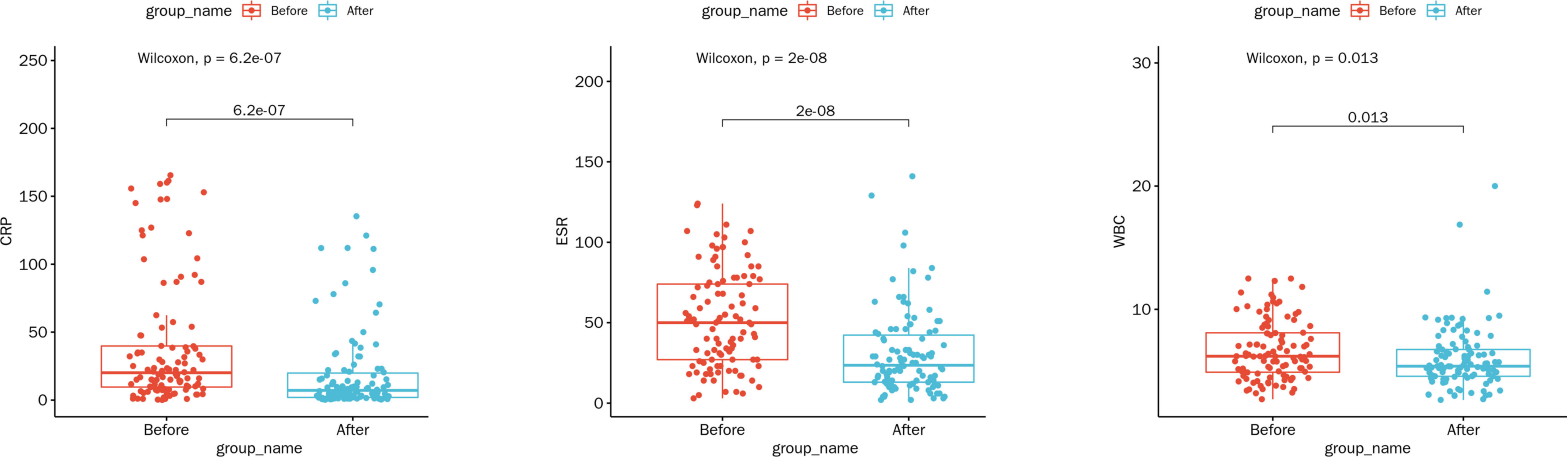
Comparison of laboratory indicators at the time of hospitalization and hospital discharge under the mNGS-guided treatment. Abbreviations: WBC, white blood cell count (10^9^/L); ESR, erythrocyte sedimentation rate (mm/h); CRP, C-reactive protein (mg/L).

At the time of hospital discharge, the CRP, ESR, and WBC of patients all significantly decreased compared with hospital admission (all *p* < 0.05; **Figure 5**). Of the 108 patients, 17 (15.74%) were completely recovered, 79 (73.15%) had improved symptoms, 12 (11.11%) voluntarily discharged from hospital, and no deaths occurred, suggesting that the patients treated with the mNGS-guided antimicrobial therapy mostly showed good outcomes.

## Discussion

As a novel diagnostic tool widely used in the field of infectious diseases, mNGS has been proven to be a powerful molecular technique over the CMTs in the field of bone and joint infections (Huang et al., 2020). However, research on the clinical value of mNGS technology in the field of spinal infection is still very rare. Recently, a study involving 30 cases of suspected spinal infection showed a higher sensitivity of mNGS but similar specificity than the culture method (Ma et al., 2022). Currently, there are various clinical tools for diagnosing spinal infection, far more than just culture, such as PCR, T-spot, and X-pert. Many of them have even been used conventionally for a long time. Therefore, in this study, we retrospectively analyzed the clinical data of 108 patients with suspected spinal infection to comprehensively explore the diagnostic value of mNGS technology. The results indicated a higher sensitivity and specificity, as well as a larger AUC of the mNGS than the CMTs in the overall identification of bacteria, mycobacteria, and fungi, suggesting a better diagnostic accuracy of mNGS in spinal infection. Moreover, the patients presented significantly decreased CRP, ESR, and WBC levels at hospital discharge, and most of them had good outcomes, further highlighting the potential value of the mNGS-guided antimicrobial therapy.

Nearly all known pathogens from clinical samples can be detected simultaneously using mNGS (Chiu and Miller, 2019; Gu et al., 2019; Gu et al., 2021). Our results further demonstrated the performance of mNGS in the detection of nearly all common pathogens that may cause spinal infection. In addition, novel pathogens including NTM (n = 5), fungi (n = 4), and bacteria (n = 15) were discovered by mNGS, suggesting a certain potential of mNGS in identifying novel pathogens associated with spinal infection. Gene Xpert, a rapid automated molecular test, is an efficient assay for the rapid diagnosis of spinal TB (Upadhyay et al., 2020; Karthek et al., 2021). A previous study showed that mNGS combined with Xpert had an overall superior advantage over conventional methods and significantly improved the etiological diagnosis of MTB (Zhou et al., 2019). In this study, however, Xpert was classified to the CMTs. Our results still showed comparable diagnostic abilities of mNGS with CMTs in the identification of MTB, including MTB identified in four cases using mNGS alone and that detected in four cases using CMTs alone. It has been acknowledged that mycobacteria require significant cell wall disruption to efficiently lyse the organisms for nucleic acid release (Simner et al., 2018). Therefore, it was speculated that the four cases of CMT-positive alone missed by mNGS might result from the low concentration of nucleic acid extraction. Two of the four cases were PCR-positive for MTB, suggesting that the minimum nucleic acid concentration required for a positive metagenomic test might be higher than that for a positive PCR test. In addition, mNGS was found to have huge advantages over the CMTs in the detection of NTM, which might be attributed to the inability of CMTs to distinguish between MTB and NTM. Generally, NTM is equally hard to culture successfully to MTB in clinical settings, and almost no specific immunological tests for NTM have been widely used yet; thus, accurate diagnosis of NTM becomes a big challenge. In our study, the NTM in five cases was identified by mNGS because of its ability to identify pathogens at the level of species but all missed by the CMTs. Moreover, MTB and NTM were detected in only two cases through biopsy pathology but missed by both mNGS and CMTs, indicating that mNGS may have a breakthrough in the diagnosis of spinal infection caused by mycobacterium. It is recommended to combine mNGS and all the CMTs for routine detection if patients are highly suspected of mycobacterium infection.

A major strength of our study was that it preliminarily and comprehensively evaluated the diagnostic value of mNGS in patients with spinal infection caused by several dominant pathogens and the patients’ outcomes under the mNGS-guided antimicrobial therapy. Although it was a retrospective study, the sample size was relatively larger compared with a previous study (Ma et al., 2022), leading to more reliability of our results. Nevertheless, there were several limitations that should be concerned. First, only four cases were ultimately diagnosed as fungal infection, leading to highly serendipitous diagnostic results. Second, a control group was not included for analysis of the outcomes; it only observed the difference before and after treatment in patients receiving mNGS-guided medication. In the future, more large-scale and well-designed studies should be performed to further explore the application value of mNGS technology in the field of spinal infection.

In conclusion, mNGS has a better diagnostic accuracy in pathogenic identification of patients with suspected spinal infection, and patients treated with NGS-guided antimicrobial therapy mostly seem to have good outcomes. In the future, more large-scale and well-designed studies should be performed to further validate our results.

## Data availability statement

The data presented in the study are deposited in the NCBI GenBank repository (accession number 2627724).

## Ethics statement

The studies involving human participants were reviewed and approved by The Institutional Review Board of Shandong Chest Hospital. Written informed consent to participate in this study was provided by the participants’ legal guardian/next of kin.

## Author contributions

LX contributed to study conception and study design and wrote the first draft of the manuscript. ZZ participated in writing “Materials and methods”. YW and CS were responsible for data acquisition, analysis, and interpretation. HT contributed to editing and reviewing the manuscript. All authors approved the submitted version.

## Acknowledgments

We thank Mrs. Furong Du, Mr. Xing Zhang, and Mr. Guanghua Lu (State Key Laboratory of Translational Medicine and Innovative Drug Development, Jiangsu Simcere Diagnostics Co., Ltd.) for their great contributions to the data collection, analysis, and interpretation of the manuscript.

## Conflict of interest

Authors YW and CS is employed by Nanjing Simcere Medical Laboratory Science Co., Ltd and Jiangsu Simcere Diagnostics Co., Ltd.

The remaining authors declare that the research was conducted in the absence of any commercial or financial relationships that could be construed as a potential conflict of interest.

## Publisher’s note

All claims expressed in this article are solely those of the authors and do not necessarily represent those of their affiliated organizations, or those of the publisher, the editors and the reviewers. Any product that may be evaluated in this article, or claim that may be made by its manufacturer, is not guaranteed or endorsed by the publisher.
